# The Spontaneous Vesicle–Micelle Transition in a Catanionic Surfactant System: A Chemical Trapping Study

**DOI:** 10.3390/molecules28166062

**Published:** 2023-08-15

**Authors:** Qihan Sun, Jiani Gong, Yujia Sun, Yao Song, Changyao Liu, Baocai Xu

**Affiliations:** Department of Daily Chemical Engineering, Beijing Technology and Business University, No. 11 Fucheng Road, Beijing 100048, China; sunqihan1999@163.com (Q.S.); gongjiani_jennifer@126.com (J.G.); 13520372492@163.com (Y.S.); xuelangyixue@163.com (Y.S.); xubaoc@btbu.edu.cn (B.X.)

**Keywords:** catanionic surfactants, chemical trapping, vesicles, interface, aggregates

## Abstract

Typically, the formation of vesicles requires the addition of salts or other additives to surfactant micelles. However, in the case of catanionic surfactants, unilamellar vesicles can spontaneously form upon dilution of the micellar solutions. Our study explores the intriguing spontaneous vesicle-to-micelle transition in catanionic surfactant systems, specifically cetyltrimethyl ammonium bromide (CTAB) and sodium octylsulfonate (SOS). To gain insights into the changes occurring at the interface, we employ a chemical trapping method to characterize variations in the molarities of sulfonate headgroups, water, and bromide ions during the transition. Our findings reveal the formation of ion pairs between the cationic component of CTAB and the anionic component of SOS, leading to tight interfacial packing in CTAB/SOS solutions. This interfacial packing promotes vesicle formation at low surfactant concentrations. Due to the significant difference in critical micelle concentration (cmc) between CTAB and SOS, an increase in the stoichiometric surfactant concentration results in a substantial rise in the SOS-to-CTAB ratio within the interfacial region. This enrichment of SOS in the aggregates triggers the transition from vesicles to micelles. Overall, our study may shed new light on the design of morphologies in catanionic and other surfactant systems.

## 1. Introduction

Above the critical micelle concentration (cmc), surfactant monomers minimize interactions with water by self-assembling into aggregates [1,2,3]. These aggregates are typically spherical micelles consisting of 30–100 or more monomers. As concentrations rise beyond the cmc, surfactants can form larger aggregates such as vesicles, rods, tubes, and ribbons [4]. The packing parameter [5], *p* = V/l * a (V: volume of the hydrophobic part, l: hydrophobic chain length, a: cross-sectional area of surfactant headgroup), offers insight into the relationship between surfactant shape and aggregate structure. Surfactants with *p* values < 1/3, 1/3–1/2, and 1/2–1 typically form spherical micelles, rod-like micelles, and vesicles, respectively. However, this parameter does not account for structural transitions with varying surfactant concentrations and other solution compositions.

In 1989, Kaler et al. discovered that anionic and cationic surfactants, when mixed in specific proportions, could spontaneously form thermodynamically stable vesicles in aqueous solutions [6]. This breakthrough sparked an intriguing research field, shedding light on important phenomena within surfactant science. The mixing ratio between anionic and cationic surfactants plays a crucial role in altering the charge and spontaneous curvature of aggregates, resulting in a diverse array of structures. Mixtures of anionic and cationic surfactants, especially with different surfactant chain lengths, exhibit complex phase diagrams encompassing micellar, vesicular, lamellar, and rod-like structures [6,7,8,9,10,11,12]. Sarkar et al. [13] conducted a study on the formation of micelles and vesicles in aqueous solutions using cetyltrimethyl ammonium bromide (CTAB) and 1-butyl-3-methylimidazolium octyl sulfate. They observed that the size of the aggregates increased with increasing CTAB volume fraction, reaching a maximum value within the range of CTAB volume fraction of 0.4–0.6. Zhao et al. [14] investigated the properties of wormlike micelles formed by mixed cationic and anionic Gemini surfactants, revealing that intermolecular hydrogen bonding played a vital role as a directional driving force, particularly facilitating the construction of long wormlike micelles. Kaler et al. [15] studied the phase behavior of a mixed system composed of sodium dodecylbenzenesulfonate and hexadecyl trimethylammonium tosylate. They discovered that it was the combination of anionic and cationic surfactants that caused the spontaneous formation of vesicles.

Vesicles are structures characterized by their spherical or elliptical shape, composed of one or more bilayers with internal cavities formed by amphiphiles [16]. They have found extensive applications as encapsulation and delivery systems [17,18,19,20,21,22]. While the formation of vesicles typically requires the addition of salts or other additives into surfactant micellar solutions [23,24,25], there are cases where vesicles spontaneously form upon dilution of the micellar phase of catanionic surfactants. In other words, as the surfactant concentration increases, a transition from vesicles to micelles occurs. This vesicle–micelle transition, contradicting conventional wisdom that added salt screens, headgroup repulsions, and reduces surface curvature, presents an interesting phenomenon. Furthermore, the concentration of surfactants in the vesicle system is generally high, and certain surfactants can be irritating to the gastrointestinal mucosa or even exhibit chronic toxicity to the human body. The formation of catanionic surfactant vesicles at relatively low concentrations could potentially enhance their application.

Currently, predicting the formation of vesicles and controlling surfactant aggregate morphologies remains a challenging yet intriguing task. Alongside hydrophobic effects driving surfactant self-assembly, multiple interactions at the interfacial region between water and aggregates play a crucial role in controlling morphologies [26]. For instance, our previous studies have revealed that specific ion pairing between headgroups and counterions in the interfacial region governs the morphological transitions in both cationic and anionic surfactant solutions [27,28]. However, it is important to note that the interfacial compositions in catanionic surfactant solutions have not been previously characterized. Our knowledge of the changes occurring in the interfacial composition of anionic headgroups, anionic counterions (to cationic headgroups), and water during the vesicle–micelle transitions in catanionic surfactant solutions is still limited. Further research is essential to gain a comprehensive understanding of these dynamics. Although numerous techniques [29,30,31,32] have been utilized to characterize the morphology of aggregates, there is a scarcity of experimental methods available for investigating the interfacial region between aggregates and solvents. To date, the chemical trapping (CT) method outlined in Section Logic of the CT Method for CTAB/SOS Aggregates stands as the singular experimental technique capable of providing estimations for the concentrations of water, anionic headgroups, halide ions, and other species within the interfacial region of aggregates.

In this study, we have investigated the vesicle–micelle transition within the catanionic surfactant system comprising CTAB and sodium octylsulfonate (SOS). In addition to analyzing the sizes and morphologies of the aggregates, we employed the CT method to assess changes in interfacial compositions during the vesicle–micelle transitions. By examining the structure, composition, and packing of the aggregates, our findings offer valuable insights into the relationship between these factors and the average compositions of the aggregate interfaces. This research contributes to a deeper understanding of the behavior and properties of catanionic surfactant systems.

### Logic of the CT Method for CTAB/SOS Aggregates

The CT method [25,26,33,34,35,36,37,38] is a distinctive approach that has been utilized to determine the interfacial compositions of surfactant aggregates. Here, we provide a concise rationale behind the CT method, while additional information can be found in other published works.

**Figure 1:**
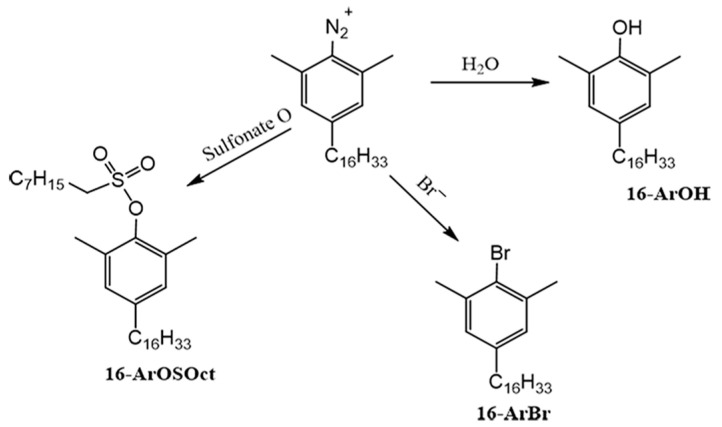
The competitive reactions between water, sulfonate headgroups, and bromide ions generate CT reaction products from the probe.

The CTAB/SOS aggregate solutions were supplemented with the long-chain diazonium probe (16-ArN_2_^+^). These probe molecules position themselves within the interfacial region, where they engage in reactions with weakly nucleophilic species such as water, sulfonate headgroups, and bromide ions. Figure 1 provides a summary of the CT reaction pathways involving the probe in CTAB/SOS aggregates. The probe’s selectivity towards these species is evaluated based on similar reactions observed with a short-chain probe (1-ArN_2_^+^) in reference solutions. Consequently, the interfacial molarities of water (H_2_O_m_), bromide ions (Br_m_), and sulfonate headgroups (SO_3m_) were measured, along with the corresponding product yields %16-ArOH, %16-ArBr, and %16-ArOSOct, respectively.

## 2. Results and Discussion

### 2.1. The Spontaneous Vesicle–Micelle Transition in CTAB/SOS Aqueous Solutions

**Figure 2:**
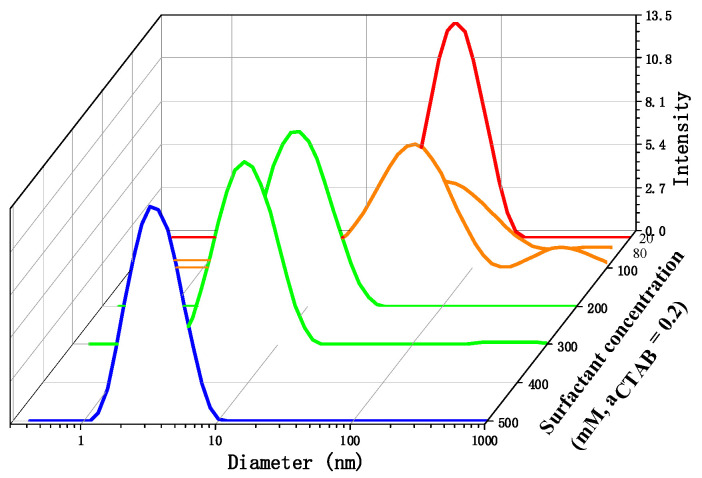
The DLS results of 20–500 mM CTAB/SOS aqueous solution (CTAB:SOS = 2:8). The different colors correspond to the different surfactant concentration.

The molar ratio between CTAB and SOS was set at 2:8. The surfactant concentration was determined by the combined concentration of CTAB and SOS ([CTAB] + [SOS]). Figure 2 illustrates the DLS results obtained from aqueous solutions of CTAB/SOS ranging from 20 to 500 mM at 25 °C. At 20 mM surfactant concentration, the peak appears at approximately 50–100 nm. As the surfactant concentration increases, the aggregate diameter decreases. In the case of the 500 mM CTAB/SOS solution, the maximum peak is observed at approximately 3–4 nm. These DLS results suggest a concentration-dependent transition from vesicles to micelles in the current catanionic system.

**Figure 3:**
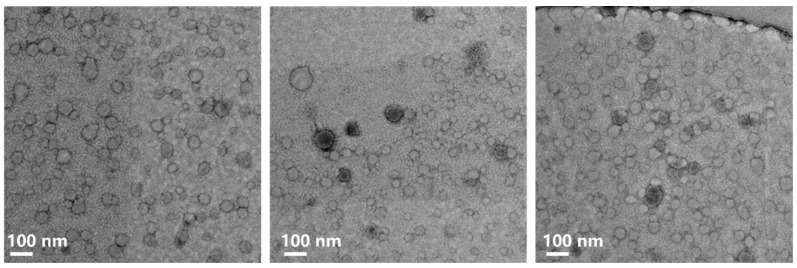
The TEM graphs of 20 mM CTAB/SOS aqueous solution.

TEM was utilized to provide additional evidence of vesicle formation at a concentration of 20 mM. Figure 3 displays representative TEM images of the 20 mM CTAB/SOS aqueous solution, clearly demonstrating the presence of vesicles whose sizes align with the DLS findings. Consequently, the combined results from DLS and TEM support the occurrence of a vesicle–micelle transition in the CTAB/SOS solution. The micropolarity of the CTAB/SOS aggregate membranes was characterized using DPH as a probe [38], assessing their anisotropic parameter (r). Higher r values are indicative of increased membrane rigidity. The obtained r values for 20 mM, 80 mM, and 100 mM CTAB/SOS aggregates were 0.11, 0.16, and 0.14, respectively. These values fell within the typical range of r values (~0.10–0.25) reported for conventional vesicle systems [31,39,40]. Notably, the r value of 500 mM CTAB/SOS aggregates was 0.088, consistent with the presence of micelles. Previous studies have demonstrated that mixed catanionic surfactants with equal chain lengths (e.g., DTAB and SDS) [41] tend to form crystalline precipitates or multilamellar structures in their aqueous solutions. In contrast, catanionic surfactants composed of surfactants with asymmetric chain lengths have a tendency to form unilamellar vesicles [8]. In our case, CTAB has a chain length twice that of SOS, which is consistent with previous literature findings.

### 2.2. Interfacial Molarity Changes during the Vesicle–Micelle Transition

In order to enhance our comprehension of the vesicle–micelle transition in the CTAB/SOS aqueous solution, we utilized the CT method to examine the variations in interfacial water (H_2_O_m_), sulfonate headgroup (SO_3m_), and bromide ion (Br_m_) molarities. The investigation involved 20–500 mM CTAB/SOS solutions with increasing stoichiometric total surfactant concentration, while maintaining a molar ratio of 2:8 between CTAB and SOS. The obtained results are presented in Table 1, which displays the HPLC peak areas, observed product yields, and normalized product yields for the dediazoniation of 16-ArN_2_^+^ in the 20–500 mM CTAB/SOS solutions containing 1 mM HBr at 25 °C. The addition of HBr was intended to minimize by-product formation. For comparative purposes, CT experiments were also conducted by replacing CTAB with NaBr at the same molarity. Unlike the CTAB/SOS catanionic system, no aggregates were formed at low surfactant concentrations in the SOS/NaBr system. Therefore, the experiments were conducted at a relatively high concentration range ([NaBr] + [SOS] ≥ 200 mM). Similar HPLC results for 200–500 mM SOS/NaBr solutions are summarized in Table 2.

The interfacial molarities of water (H_2_O_m_), sulfonate headgroups (SO_3m_), and bromide ions (Br_m_) in CTAB/SOS or NaBr/SOS aqueous solutions in surfactant aggregates were determined based on the normalized yields of 16-ArOH_N_, 16-ArOSOct_N_, and 16-ArBr_N_, respectively. Calculations of the total observed yields and normalized yields of 16-ArOH_N_, 16-ArOSOct_N_, and 16-ArBr_N_ are summarized in the Supplementary Material. In the case of CTAB/SOS, as the total surfactant concentration increased from 20 to 500 mM, the interfacial molarities of sulfonate headgroups increased from 1.9 to 2.6 M, while the interfacial water molarity decreased from 34 to 27 M. The change in interfacial bromide molarity, ranging from 0.05 to 0.08 M, was relatively small. For 200–500 mM NaBr/SOS solutions, the interfacial molarities of sulfonate headgroups, water, and bromide ions were approximately 1.3–1.4 M, 38–39 M, and 0.06–0.07 M, respectively. The detailed calculations are listed in the Supplemental Material and the numeric values of the interfacial molarities are listed in Tables S2 and S3.

### 2.3. Discussion on the Interfacial Molarities and the Vesicle–Micelle Transition in CTAB/SOS Solutions

From a Gibbs phase rule perspective, aqueous solutions of CTAB/SOS consist of five components, including CTA^+^OS^−^, Na^+^Br^−^, CTA^+^Br^−^, Na^+^OS^−^, and water. In comparison to bromide ions, the anionic portion of SOS exhibits a stronger tendency to enter the interfacial region of aggregates and form ion pairs with CTA^+^. As a result, a significant amount of bromide ions are expelled into the bulk water phase, leading to an extremely low value of Br_m_. Instead of functioning as counterions for CTAB, they can be considered as co-ions, similar to the NaBr/SOS systems. Our findings, which demonstrate similar Br_m_ values in both systems, provide support for the aforementioned concept. Additionally, the formation of ion pairing between CTA^+^ and OS^−^ leads to the transfer of interfacial water into the bulk water phase. Therefore, the interfacial hydration in the NaBr/SOS system was significantly higher compared to the CTAB/SOS system.

**Figure 4:**
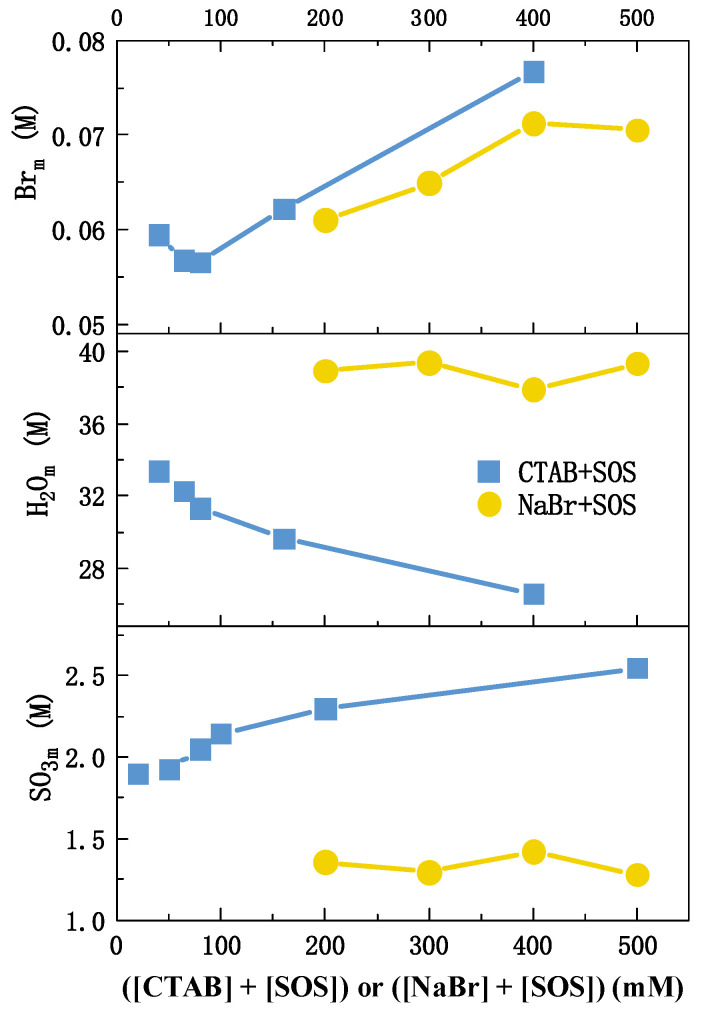
Interfacial molarities of water (H_2_O_m_), sulfonate headgroups (SO_3m_), and bromide ions (Br_m_) versus total stoichiometric concentration of [CTAB] + [SOS] or [NaBr] + [SOS].

It is intriguing to observe that the interfacial sulfonate headgroup molarity in CTAB/SOS aggregates is significantly higher than that in SOS/salt aggregates, despite SOS being the sole surfactant present in the SOS/salt aggregates, whereas two different surfactants are involved in CTAB/SOS aggregates. Assuming no synergistic effect between the two surfactants in the CTAB/SOS system, the addition of CTAB should only dilute the arrangement of SOS in the aggregates. In other words, the interfacial molarity of SO_3m_ in the SOS/NaBr aggregates should be higher than the corresponding value in CTAB/SOS aggregates. Surprisingly, as depicted in Figure 4, at the same stoichiometric SOS concentration, the interfacial molarity of SOS headgroups in the CTAB/SOS system is actually approximately twice that in the SOS/NaBr system. Therefore, although the CT probe does not directly react with the cationic headgroups of CTAB to determine the arrangement of CTAB at the interface, it can be inferred that the surfactant arrangement in the CTAB/SOS system is much more compact than that in the SOS/NaBr system. Consequently, vesicles are formed at very low concentrations in CTAB/SOS aqueous solutions.

**Figure 5:**
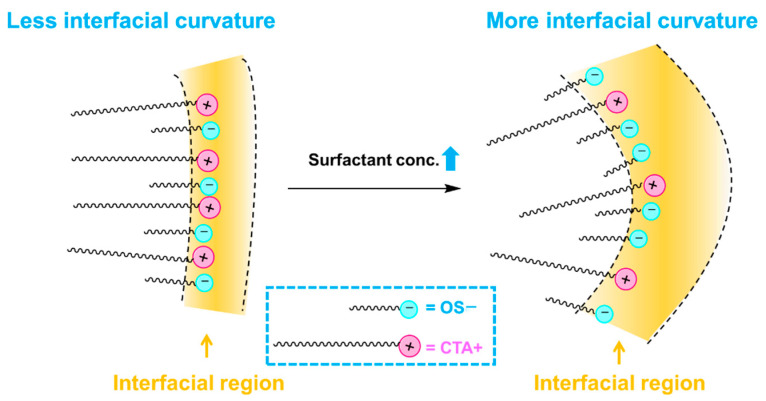
As the surfactant concentration increases, more SOS molecules enter the aggregates. This increase in the ratio of SOS in the interfacial region dilutes the tight packing of CTAB/SOS observed at low concentrations, leading to a higher curvature.

While it is noteworthy that the stoichiometric concentration of SOS is four times that of CTAB, it is important to consider that the molar ratio of SOS and CTAB within the aggregates might not be equivalent. Because of the significant difference in chain length between CTAB and SOS, as well as the considerably higher cmc of SOS (130–153 mM) [42] compared to CTAB (0.88 mM) [8], CTAB exhibits a higher tendency to enter the aggregates. At low surfactant concentrations, the interfacial molarities of CTAB and SOS are conducive to vesicle formation. However, as the surfactant concentration increases, more SOS molecules enter the aggregates. This increase in the ratio of SOS in the interfacial region dilutes the tight packing of CTAB/SOS observed at low concentrations, leading to a higher curvature (as depicted in Figure 5). Consequently, the vesicles transition into micelles at relatively high surfactant concentrations.

**Figure 6:**
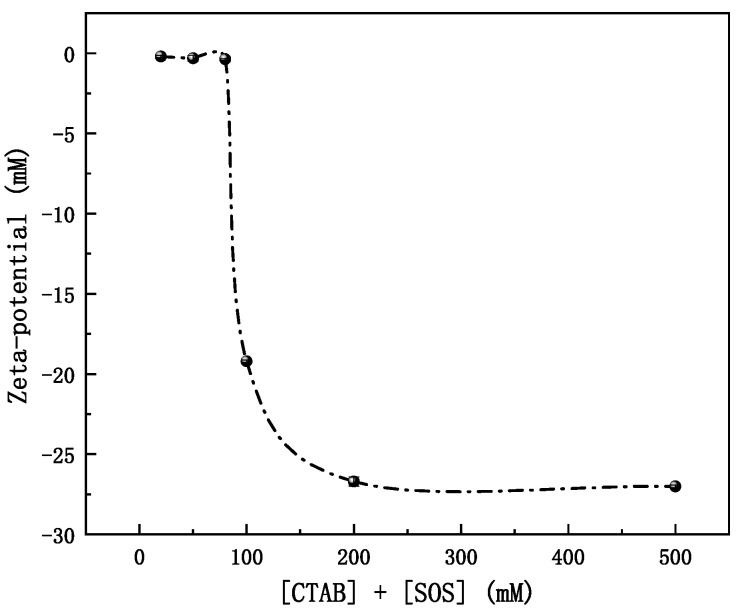
Zeta-potential versus total stoichiometric concentration of [CTAB] + [SOS].

Figure 6 illustrates our zeta-potential findings, which align with the preceding discussion. Within a total stoichiometric surfactant concentration range of 20 to 80 mM, the zeta-potential remains relatively stable at approximately −0.3 mV. This stability suggests an imperfect charge neutralization in the interfacial region of CTAB/SOS vesicles. As the stoichiometric surfactant concentration increases from 100 to 500 mM, the zeta-potential becomes progressively more negative, approaching −27 mV. These outcomes indicate that, even though the SOS/CTAB ratio remains constant in the bulk solution, the SOS-to-CTAB ratio at the interfacial region gradually increases with rising stoichiometric surfactant concentration. In the lower concentration range, the near-zero zeta-potential value signifies tight packing in the interfacial region, contributing to vesicle formation. Despite the general notion that low zeta-potential correlates with low stability in aggregates [43], this is not universally accurate. Our results are in line with the findings of the Abe group [44], which demonstrate that DDAB-SDS vesicles with very low zeta-potential exhibit greater stability than highly charged DDAB vesicles.

## 3. Materials and Methods

### 3.1. Materials

Cetyltrimethyl ammonium bromide (CTAB, 99%), sodium octylsulfonate (SOS, 98%), and sodium bromide (99%) were obtained from Macklin Biochemical Co., Ltd., Hebei, China and used as received. The probe, 16-ArN_2_^+^, and dediazoniation products were prepared previously in the lab.

### 3.2. Dynamic Light Scattering (DLS) Measurements

DLS measurements were performed utilizing a Malvern Zetasizer Nano ZS instrument equipped with a solid-state He-Ne laser (λ = 632.8 nm) with a power output of 22 mW. The scattering angle employed for the measurements was set at 173°.

### 3.3. Transmission Electron Microscopy (TEM) Measurements

TEM micrographs of negatively stained samples were obtained using an Oxford X-MAX JEM-2100 transmission electron microscope operating at 120 kV voltage. The staining agent employed was a 2% aqueous solution of uranyl acetate.

### 3.4. Steady-State Fluorescence Measurements

The anisotropic parameter (r) was determined using a steady-state/transient fluorescence spectrometer (FLS1000, Edinburgh, UK). A 5 μL DPH methanol solution was extracted from the sample bottle, air-dried at room temperature in a fume hood, and mixed with the sample solution, resulting in a final DPH concentration of 1 μM. The fluorescence excitation wavelength was set at 350 nm, while the fluorescence emission range spanned 380 to 550 nm. Both the excitation and emission slits were adjusted to 2 nm, and an integration time of 1 s was employed. The fluorescence emission intensity at 450 nm was utilized to calculate the r value using Equations (1) and (2).

(1) $$r=\frac{I_{VV}-{GI}_{VH}}{I_{VV}+2{GI}_{VH}}$$

(2) $$G=I_{HV}/I_{HH}$$

where I_VH_ and I_VV_ are the respective fluorescence intensity emitted by horizontal and vertical polarization, respectively, when the polarizer is excited in the vertical direction. I_HV_ and I_HH_ are the fluorescence intensities in the horizontal direction of the excited polarizer. G as a grating correction factor is the ratio of I_HV_ to I_HH_.

### 3.5. Zeta-Potential Measurements

Utilize the ZetaSizer Nano potential particle size analyzer for determining the zeta-potential of the surfactant solutions. Ensure that the temperature is set to stabilize at 25 ± 0.1 °C. Perform six parallel measurements for each sample solution.

### 3.6. Chemical Trapping Experiments in CTAB/SOS Aqueous Solutions

The chemical trapping reactions were conducted according to the previously reported method, allowing them to proceed for 48 h at a temperature of 25 °C [26,37]. HPLC measurements were performed using an Agilent Technologies 1200 series instrument from Santa Clara, California, USA, which included a UV/Vis detector, a ZORBAX Eclipse Plus C18 column, and 1.1.143.0 Agilent Chemstation software (accessed on 18 July 2023). The percentage yields were determined by calculating the average peak areas from triplicate injections and referring to the appropriate calibration curves.

## 4. Conclusions

The vesicle–micelle transition in CTAB/SOS (2:8) aqueous solutions was characterized using DLS and TEM. The CT method was employed to estimate the interfacial molarities of sulfonate headgroups, water, and bromide ions. The results confirmed the formation of ion pairing between CTA^+^ and OS^−^, as evidenced by the interfacial molarity of bromide ions and water. Interestingly, at a fixed SOS stoichiometric concentration, the interfacial sulfonate headgroup molarity in CTAB/SOS was significantly higher than that in SOS/salt aggregates, indicating a tight interfacial packing in CTAB/SOS solutions that facilitates vesicle formation at low surfactant concentrations. Due to the notable difference in cmc between CTAB and SOS, the molar ratio between them in the interfacial region differed from that in the bulk phase. As the surfactant concentration increased, the ratio of SOS to CTAB in the interfacial region also increased, disrupting the ideal packing between CTAB and SOS. This resulted in an enrichment of SOS in aggregates and triggered the vesicle–micelle transition. Vesicles have gained extensive usage in various fields, including molecular recognition, drug delivery, nanosynthesis, and as reliable models for simulating biological membranes. The formation of vesicles at lower surfactant concentrations offers the advantage of reduced toxicity and cost. The interfacial molarity findings of the reversed micelle–vesicle transition in catanionic surfactant contribute to a deeper understanding of the relationship between structure and interfacial compositions.

## Figures and Tables

**Table 1:** HPLC peak areas and observed and normalized product yields for dediazoniation reaction of 16-ArN_2_^+^ in solutions of 20–500 mM CTAB/SOS at 25 °C.

[CTAB] + [ SOS]	Peak Areas (10^2^ mAU·s)			Observed Yields (%)				Normalized Yields (%)		
(mM)	16-ArOH	16-ArOSOct	16-ArBr	16-ArOH	16-ArOSO_ct_	16-ArBr	Total	16-ArOH_N_	16-ArOSOct_N_	16-ArBr_N_
20	34.75	10.37	0.11	77.4	15.8	2.0	95.1	81.3	16.6	2.1
20	33.80	10.76	0.11	75.2	16.4	2.0	93.5	80.4	17.5	2.1
50	29.21	9.16	0.15	70.2	15.1	2.2	87.4	80.2	17.3	2.5
50	29.51	9.26	0.16	70.9	15.3	2.2	88.4	80.2	17.3	2.5
80	30.53	10.39	0.19	73.4	17.1	2.2	92.8	79.1	18.5	2.4
80	30.60	10.31	0.18	73.6	17.0	2.2	92.8	79.3	18.3	2.4
100	30.52	11.01	0.19	73.4	18.2	2.2	93.8	78.3	19.4	2.4
100	30.50	10.86	0.19	73.3	17.9	2.2	93.5	78.4	19.2	2.4
200	29.22	11.50	0.31	70.2	19.0	2.4	91.5	76.7	20.7	2.6
200	29.14	11.31	0.29	70.0	18.7	2.3	91.0	76.9	20.5	2.6
500	28.88	13.00	0.75	69.3	21.5	2.9	93.7	74.0	22.9	3.1
500	28.90	13.01	0.74	69.4	21.5	2.9	93.7	74.0	22.9	3.1

**Table 2:** HPLC peak areas and observed and normalized product yields for dediazoniation reaction of 16-ArN_2_^+^ in solutions of 200–500 mM NaBr/SOS at 25 °C.

[NaBr] + [ SOS]	Peak Areas (10^2^ mAU·s)			Observed Yields (%)				Normalized Yields (%)		
(mM)	16-ArOH	16-ArOSOct	16-ArBr	16-ArOH	16-ArOSOct	16-ArBr	Total	16-ArOH_N_	16-ArOSOct_N_	16-ArBr_N_
200	29.95	6.24	0.13	80.1	11.5	2.4	94.0	85.3	12.2	2.5
200	30.26	6.25	0.13	81.0	11.5	2.4	94.8	85.4	12.1	2.5
200	30.41	6.26	0.14	81.4	11.5	2.4	95.3	85.4	12.1	2.5
300	30.19	5.95	0.23	80.8	10.9	2.5	94.3	85.7	11.6	2.7
300	30.27	5.95	0.23	81.0	10.9	2.5	94.5	85.8	11.6	2.7
300	30.41	5.96	0.22	81.4	10.9	2.5	94.9	85.8	11.5	2.6
400	29.78	6.54	0.40	79.7	12.0	2.8	94.4	84.4	12.7	2.9
400	29.85	6.55	0.37	79.9	12.0	2.7	94.6	84.4	12.7	2.9
400	29.87	6.55	0.40	79.9	12.0	2.7	94.7	84.4	12.7	2.9
500	29.88	5.81	0.34	79.9	10.7	2.7	93.3	85.7	11.4	2.9
500	29.88	5.82	0.35	79.9	10.7	2.7	93.3	85.7	11.4	2.9
500	29.98	5.83	0.34	80.2	10.7	2.7	93.6	85.7	11.4	2.9

## Data Availability

The data presented in this study are available on request from the corresponding authors.

## Supplementary Materials

The following supporting information can be downloaded at: https://www.mdpi.com/article/10.3390/molecules28166062/s1. Figure S1: The plot of the selectivity between bromide ion and water, S_w_^Br^ versus [Br_t_] in TMABr aqueous solutions; Table S1: Calibration curves for observed product yields; Table S2: Estimated values of interfacial molarities of Br_m_, H_2_O_m_, and SO_3m_ in aqueous solutions of 20–500 mM CTAB/SOS solutions at 25 °C. [HBr] = 1 mM; Table S3: Estimated values of interfacial molarities of Br_m_, H_2_O_m_, and SO_3m_ in aqueous solutions of 200–500 mM NaBr/SOS solutions at 25 °C. [HBr] = 1 mM.

## Sample Availability

Samples of the compounds are available from the authors.

## References

[1] Romsted L.S., Gale P., Steed J. (2012). Introduction to Surfactant Self-Assembly. Encyclopedia of Supramolecular Chemistry: From Molecules to Nanomaterials.

[2] Menger F.M. (2002). Supramolecular Chemistry And Self-assembly Special Feature: Supramolecular chemistry and self-assembly. Proc. Natl. Acad. Sci. USA.

[3] Rosen M.J., Kunjappu J.T. (2012). Surfactants and Interfacial Phenomena.

[4] Ghosh S., Ray A., Pramanik N. (2020). Self-assembly of surfactants: An overview on general aspects of amphiphiles. Biophys. Chem..

[5] Israelachvili J.N., Mitchell D.J., Ninham B.W. (1976). Theory of self-assembly of hydrocarbon amphiphiles into micelles and bilayers. J. Chem. Soc. Faraday Trans..

[6] Kaler E.W., Murthy A.K., Rodriguez B.E., Zasadzinski J.A.N. (1989). Spontaneous Vesicle Formation in Aqueous Mixtures of Single-Tailed Surfactants. Science.

[7] Jung H.T., Coldren B., Zasadzinski J.A., Iampietro D.J., Kaler E.W. (2001). The origins of stability of spontaneous vesicles. Proc. Natl. Acad. Sci. USA.

[8] Kawasaki H., Imahayashi R., Maeda H. (2002). Effects of hydrophobic counterions on the phase behavior of tetradecyldimethylhydroxylammonium chloride in aqueous solutions. Langmuir.

[9] Li S.-J., Lai L., Mei P., Li Y., Cheng L., Ren Z.-H., Liu Y. (2018). Equilibrium and dynamic surface properties of cationic/anionic surfactant mixtures based on bisquaternary ammonium salt. J. Mol. Liq..

[10] Yatcilla M.T., Herrington K.L., Brasher L.L., Kaler E.W., Chiruvolu S., Zasadzinski J.A. (1996). Phase Behavior of Aqueous Mixtures of Cetyltrimethylammonium Bromide (CTAB) and Sodium Octyl Sulfate (SOS). Phys. Chem..

[11] Silva O.F., de Rossi R.H., Correa N.M., Silber J.J., Falcone R.D. (2018). Spontaneous catanionic vesicles formed by the interaction between an anionic beta-cyclodextrins derivative and a cationic surfactant. RSC Adv..

[12] Tondre C., Caillet C. (2001). Properties of the amphiphilic films in mixed cationic/anionic vesicles: A comprehensive view from a literature analysis. Adv. Colloid Interface Sci..

[13] Ghosh S., Ghatak C., Banerjee C., Mandal S., Kuchlyan J., Sarkar N. (2013). Spontaneous Transition of Micelle-Vesicle-Micelle in a Mixture of Cationic Surfactant and Anionic Surfactant-like Ionic Liquid: A Pure Nonlipid Small Unilamellar Vesicular Template Used for Solvent and Rotational Relaxation Study. Langmuir.

[14] Pei X., Zhao J., Wei X. (2011). Wormlike micelles formed by mixed cationic and anionic gemini surfactants in aqueous solution. J. Colloid Interface Sci..

[15] Kaler E.W., Herrington K.L., Murthy A.K., Zasadzinski J.A.N. (1992). Phase behavior and structures of mixtures of anionic and cationic surfactants. Phys. Chem..

[16] Fendler J.H. (1984). Membrane Mimetic Chemistry Systems that mimic aspects of biomembranes hold promise for controlling the rates and stereochemistry of reactions, enhancing solar energy conversion, and targeting drug delivery. Chem. Eng. News Arch..

[17] Costa C., Oliveira I.S., Silva J.P.N., Silva S.G., Botelho C., do Vale M.L.C., Real Oliveira M., Gomes A.C., Marques E.F. (2021). Effective cytocompatible nanovectors based on serine-derived gemini surfactants and monoolein for small interfering RNA delivery. J. Colloid Interface Sci..

[18] Rajput S.M., Kumar S., Aswal V.K., El Seoud O.A., Malek N.I., Kailasa S.K. (2018). Drug-Induced Micelle-to-Vesicle Transition of a Cationic Gemini Surfactant: Potential Applications in Drug Delivery. Chemphyschem.

[19] Tian B., Tao X., Ren T., Weng Y., Lin X., Zhang Y., Tang X. (2012). Polypeptide-based vesicles: Formation, properties and application for drug delivery. J. Mater. Chem..

[20] Zayka P., Parr B., Robichaud H., Hickey S., Topping A., Holt E., Watts D.B.E., Soto N., Stein D.C., DeShong P. (2023). Evaluating methods to create protein functionalized catanionic vesicles. Soft Matter.

[21] Zhang F., Yao Q., Chen X., Zhou H., Zhou M., Li Y., Cheng H. (2023). In-depth study of anticancer drug diffusion through a cross-linked -pH-responsive polymeric vesicle membrane. Drug Deliv..

[22] Bramer T., Dew N., Edsman K. (2007). Pharmaceutical applications for catanionic mixtures. J. Pharm. Pharmacol..

[23] Cano-Sarabia M., Angelova A., Ventosa N., Lesieur S., Veciana J. (2010). Cholesterol induced CTAB micelle-to-vesicle phase transitions. J. Colloid Interface Sci..

[24] Thapa U., Dey J., Kumar S., Hassan P.A., Aswal V.K., Ismail K. (2013). Tetraalkylammonium ion induced micelle-to-vesicle transition in aqueous sodium dioctylsulfosuccinate solutions. Soft Matter.

[25] Yao K., Sun L., Ding X., Wang Y., Liu T., Liu C., Tan J., Zhao L., Xu B., Romsted L. (2020). Simultaneous determination of interfacial molarities of an alcohol, bromide ion, and water during an alcohol induced microstructural transition: The difference between medium and long chain alcohols. Soft Matter.

[26] Romsted L.S. (2007). Do amphiphile aggregate morphologies and interfacial compositions depend primarily on interfacial hydration and ion-specific interactions? The evidence from chemical trapping. Langmuir.

[27] Liu C., Wang Y., Gao Y., Zhang Y., Zhao L., Xu B., Romsted L.S. (2019). Effects of interfacial specific cations and water molarities on AOT micelle-to-vesicle transitions by chemical trapping: The specific ion-pair/hydration model. Phys. Chem. Chem. Phys..

[28] Geng Y., Romsted L.S., Menger F. (2006). Specific ion pairing and interfacial hydration as controlling factors in gemini micelle morphology. Chemical trapping studies. J. Am. Chem. Soc..

[29] Guttman S., Ocko B.M., Deutsch M., Sloutskin E. (2016). From faceted vesicles to liquid icoshedra: Where topology and crystallography meet. Curr. Opin. Colloid Interface Sci..

[30] Zheng Z.B., Zhou M., Qiao W.H., Luo L.M. (2015). Spontaneous Vesicle Formation in Mixtures of Quaternary Ammonium Compounds with Carbamate and Sodium Dodecylbenzene Sulfonate. J. Surfactants Deterg..

[31] Xu H.F., Du N., Song Y.W., Song S., Hou W.G. (2017). Microviscosity, encapsulation, and permeability of 2-ketooctanoic acid vesicle membranes. Soft Matter.

[32] Segota S., Heimer S., Tezak D. (2006). New catanionic mixtures of dodecyldimethylammonium bromide/sodium dodecylbenzenesulphonate/water I. Surface properties of dispersed particles. Colloid Surf. A Physicochem. Eng. Asp..

[33] Chaudhuri A., Loughlin J.A., Romsted L.S., Yao J. (1993). Arenediazonium Salts: New Probes of the Interfacial Compositions of Association Colloids. 1. Basic Approach, Methods, and Illustrative Applications. Am. Chem. Soc..

[34] Dar A.A., Romsted L.S., Nazir N., Zhang Y., Gao X., Gu Q., Liu C. (2017). A novel combined chemical kinetic and trapping method for probing the relationships between chemical reactivity and interfacial H(2)O, Br(-) and H(+) ion molarities in CTAB/C(12)E(6) mixed micelles. Phys. Chem. Chem. Phys..

[35] Gong J., Yao K., Sun Q., Sun Y., Sun L., Liu C., Xu B., Tan J., Zhao L., Xu B. (2022). Interfacial Composition of Surfactant Aggregates in the Presence of Fragrance: A Chemical Trapping Study. Molecules.

[36] Sun L., Gong J., Xu B., Wang Y., Ding X., Zhang Y., Liu C., Zhao L., Xu B. (2022). Ion-Specific Effects on Vesicle-to-Micelle Transitions of an Amino Acid Surfactant Probed by Chemical Trapping. Langmuir.

[37] Sun Y., Sun Q., Sun L., Chen Z., Jiang R., Gong J., Zhang Y., Liu C., Zhao L., Xu B. (2023). Tetraalkylammonium counterion effects on lauroyl β-alanine: A chemical trapping study. Colloids Surf. A—Physicochem. Eng. Asp..

[38] Roy S., Mohanty A., Dey J. (2005). Microviscosity of bilayer membranes of some N-acylamino acid surfactants determined by fluorescence probe method. Chem. Phys. Lett..

[39] Engberg O., Nurmi H., Nyholm T.K.M., Slotte J.P. (2015). Effects of Cholesterol and Saturated Sphingolipids on Acyl Chain Order in 1-Palmitoyl-2-oleoyl-sn-glycero-3-phosphocholine Bilayers-A Comparative Study with Phase-Selective Fluorophores. Langmuir.

[40] Shinitzky M., Barenholz Y. (1974). Dynamics of the hydrocarbon layer in liposomes of lecithin and sphingomyelin containing dicetylphosphate. J. Biol. Chem..

[41] Herrington K.L., Kaler E.W., Miller D.D., Zasadzinski J.A., Chiruvolu S. (1993). Phase Behavior of Aqueous Mixtures of Dodecyltrimethylammonium Bromide (DTAB) and Sodium Dodecyl Sulfate (SDS). Phys. Chem..

[42] Annunziata O., Costantino L., D’Errico G., Paduano L., Vitagliano V. (1999). Transport properties for aqueous sodium sulfonate surfactants-2. Intradiffusion measurements: Influence of the obstruction effect on the monomer and micelle mobilities. J. Colloid. Interface Sci..

[43] Jiang Y., Li F., Luan Y., Cao W., Ji X., Zhao L., Zhang L., Li Z. (2012). Formation of drug/surfactant catanionic vesicles and their application in sustained drug release. Int. J. Pharm..

[44] Kondo Y., Uchiyama H., Yoshino N., Nishiyama K., Abe M. (1995). Spontaneous Vesicle Formation from Aqueous Solutions of Didodecyldimethylammonium Bromide and Sodium Dodecyl sulfate Mixtures. Langmuir.

