# Supplementary material for: The Spontaneous Vesicle–Micelle Transition in a Catanionic Surfactant System: A Chemical Trapping Study
Source: Molecules. 2023 Aug 15;28(16):6062. doi: 10.3390/molecules28166062 (PMC10457922; doi:10.3390/molecules28166062)
Supplement: Supplementary file 1 [file molecules-28-06062-s001.zip › molecules-2537763-supplementary.pdf]

**Supplementary Information**

# The spontaneous vesicle–micelle transition in a catanionic surfactant system: A chemical trapping study

Qihan Sun, Jiani Gong, Yujia Sun, Yao Song, Changyao Liu*, Baocai Xu

*Department of Daily Chemical Engineering, Beijing Technology and Business University, No. 11 Fucheng Road, Beijing, 100048, People's Republic of China*

*Corresponding Author.
E-mail: changyaoliu@btbu.edu.cn (C. Liu)

## Table of Contents

Section I: Chemical trapping with 1-$ArN_2^+$ in aqueous TMABr solutions S2
Section II: Equations for calculations of chemical trapping product yields and interfacial molarities S2
Section III: Interfacial molarities S3

**Section Ⅰ: Chemical trapping with 1-ArN$_2^+$ in aqueous TMABr solutions.**

**Figure S1** shows the plot of the selectivity between bromide ion and water, $S_w^{Br}$ versus [Br$_t$] in TMABr aqueous solutions. The data was also fitted into Equation S1, which was used to estimate the selectivity, $S_w^{Br}$, at the interfacial region.

$$S_W^{Br} = 11.2957 - 4.2421 \times \ln([Br_t]) \quad \text{(S1)}$$

**Figure S1** Plot between $S_w^{Br}$ and [Br$_t$], in TMABr aqueous solutions ( [HBr] = 1 mM) (a) containing 0 to 3.5 M [Br$_t$] ; and (b) containing 0.01 to 0.04 M [Br$_t$] fitted to equation $S_W^{Br} = 11.2957 - 4.2421 \times \ln([Br_t])$.

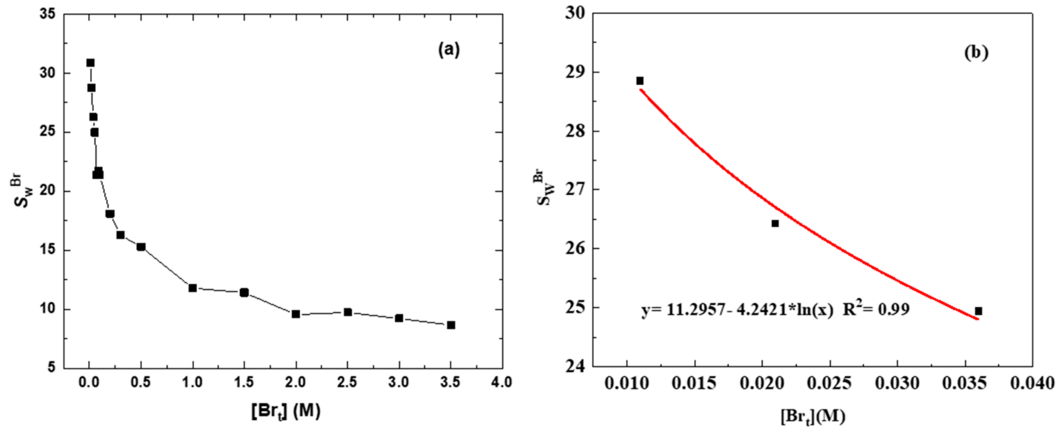


**Section II Equations for calculations of chemical trapping product yields and interfacial molarities**

**Table S1.** Calibration curves for observed product yields

| Products | Calibration curves | $R^2$ | Retention time（min） |
|---|---|---|---|
| 16-ArOH | y= 1.000 × 10$^8$x + 77.138 | 0.9995 | 13.5 |
| 16-ArBr | y= 2.000 × 10$^8$x – 160.74 | 0.9994 | 30 |
| 16-ArOSOct | y= 1.459 × 10$^8$x | 1 | 22 |

The normalized product yields were calculated by **Equation S2-S4**:

$$\%16\text{-ArOH}_N = 100 \times \frac{\%16\text{-ArOH}}{\%16\text{-ArOH}+\%16\text{-ArOSOct}+\%16\text{-ArBr}} \quad \text{(S2)}$$

$$\%16\text{-ArBr}_N = 100 \times \frac{\%16\text{-ArBr}}{\%16\text{-ArOH}+\%16\text{-ArOSOct}+\%16\text{-ArBr}} \quad \text{(S3)}$$

$$\%16\text{-ArOSOct}_N = 100 \times \frac{\%16\text{-ArOSOct}}{\%16\text{-ArOH}+\%16\text{-ArOSOct}+\%16\text{-ArBr}} \quad \text{(S4)}$$

**Equations S1 and S5-S6** were employed for interfacial molarity calculation [1]:

$$\%16\text{-ArOSOct}_N = 9.036\,[SO_{3m}] - 0.103 \quad \text{(S5)}$$

$$\%16\text{-ArOH}_N = 0.914\,[H_2O_m] + 49.72 \quad (S6)$$

**Section III: Interfacial molarities**

**Table S2** Estimated values of interfacial molarities of $Br_m$, $H_2O_m$, and $SO_{3m}$, in aqueous solutions of 20 – 500 mM CTAB/SOS solutions at 25ºC. [HBr] = 1 mM.

| [CTAB]+[SOS] | Normalized yields (%) | | | Interfacial molarities(M) | | |
|---|---|---|---|---|---|---|
| (mM) | 16-ArOH$_N$ | 16-ArOSOct$_N$ | 16-ArBr$_N$ | $H_2O_m$ | $SO_{3m}$ | $Br_m$ |
| 20 | 80.9 | 17.1 | 2.1 | 34.08 | 1.90 | 0.05 |
| 50 | 80.2 | 17.3 | 2.5 | 33.39 | 1.92 | 0.06 |
| 80 | 79.2 | 18.4 | 2.4 | 32.27 | 2.05 | 0.06 |
| 100 | 78.4 | 19.3 | 2.4 | 31.33 | 2.14 | 0.06 |
| 200 | 76.8 | 20.6 | 2.6 | 29.63 | 2.29 | 0.06 |
| 500 | 74.0 | 22.9 | 3.1 | 26.57 | 2.55 | 0.08 |

**Table S3** Estimated values of interfacial molarities of $Br_m$, $H_2O_m$, and $SO_{3m}$, in aqueous solutions of 200 – 500 mM NaBr/SOS solutions at 25ºC. [HBr] = 1 mM.

| [NaBr]+[SOS] | Normalized yields (%) | | | Interfacial molarities(M) | | |
|---|---|---|---|---|---|---|
| (mM) | 16-ArOH$_N$ | 16-ArOSOct$_N$ | 16-ArBr$_N$ | $H_2O_m$ | $SO_{3m}$ | $Br_m$ |
| 200 | 85.3 | 12.1 | 2.5 | 38.95 | 1.36 | 0.06 |
| 300 | 85.7 | 11.6 | 2.7 | 39.41 | 1.29 | 0.06 |
| 400 | 84.4 | 12.7 | 2.9 | 37.92 | 1.42 | 0.07 |
| 500 | 85.7 | 11.4 | 2.9 | 39.34 | 1.28 | 0.07 |

## Reference

[1] C. Liu, Y. Wang, Y. Gao, Y. Zhang, L. Zhao, B. Xu, L.S. Romsted, Effects of interfacial specific cations and water molarities on AOT micelle-to-vesicle transitions by chemical trapping: the specific ion-pair/hydration model, Phys. Chem. Chem. Phys., 21 (2019) 8633-8644.
